# Prevalence of Human Papillomavirus (HPV) Infection and its Association With Pap Smear Findings Among Women Attending a Gynecology Clinic in Khorasan Razavi-Iran

**DOI:** 10.34172/jrhs.2024.164

**Published:** 2024-09-30

**Authors:** Sareh Etemad, Amir Mohammad Asghari Baghalan, Bita Naeimi, Shadi Mehrzad, Saina Adib Amin, Mohammad Soudyab, Reza Jafarzadeh Esfehani

**Affiliations:** ^1^Blood Borne Infections Research Center, Academic Center for Education, Culture, and Research (ACECR), Khorasan Razavi Branch, Mashhad, Iran

**Keywords:** Human papillomavirus, Cancer, Genotype, Cervix

## Abstract

**Background:** Human papillomavirus (HPV) infection is the most common sexual transmitted disease and Pap smears and HPV testing are crucial for early detection. Advancements in HPV testing improve accuracy, but optimal screening strategies are still debated. This cross-sectional study explores HPV genotypes and predictors of infection among Iranian women undergoing gynecological screening.

**Study Design:** A retrospective cross-sectional study.

**Methods:** Women undergoing their initial cervical screening enrolled in this study. Cervical cytology samples for Pap smear analysis were collected from women referred to the genetic laboratory of Academic Center for Education Culture and Research (ACECR), Khorasan Razavi, during gynecological visits, adhering to standardized liquid-based cytology protocols. These samples were obtained over a one-year period since January 2023. Statistical analyses were conducted using SPSS version 21.0, with a significance level set at *P*<0.05.

**Results:** A total of 328 women enrolled in this study. The mean age of participants was 36±11 years and the overall HPV prevalence was 37.5% (n=123). Among HPV-positive women, nearly half (48.7%) had a single HPV genotype. Genotypes 6 (13%), 16 (12.3%), and 53 (6.7%) were the most prevalent types. Notably, high-risk HPV genotypes (16 and 18 among all) were identified in one-fourth of the study population. Women with endocervical/transformation zone cells had 25% higher odds of having HPV infection, and having mild, moderate, and severe inflammation increased the odds of having HPV infection by 14%, 11%, and 20%, respectively.

**Conclusion:** The considerably high prevalence of HPV infection highlights the significance of HPV prevention programs in this population. Neither bacterial vaginosis nor candida infection showed a direct link to HPV positivity.

## Background

 Human papillomavirus (HPV) infection is the most prevalent sexually transmitted infection (STI) globally, affecting millions of people annually. Up to now, over 200 HPV genotypes have been identified and categorized as either low-risk or high-risk based on their association with cervical cancer development.^1^ High-risk HPV genotypes, particularly types 16 and 18, are responsible for the vast majority of cervical cancer cases. Given this established link, cervical cancer screening program, which often includes HPV testing along with Pap smear cytology, plays a crucial role in early detection and prevention of cervical cancer.^2^

 Regarding the changes and introduction of novel HPV genotypes, understanding the prevalence and distribution of HPV genotypes within a population becomes critical for developing effective prevention and management strategies. While numerous studies have explored HPV epidemiology, variations exist based on geographic location, population demographics, and screening programs.^3^ Although HPV is a necessary cause for cervical cancer, not all infections progress to malignancy. The body’s immune response plays a vital role in clearing HPV infections. However, factors that impair immune function, such as HIV infection or immunosuppressive medications, can increase the risk of persistent HPV infection and subsequent cervical cancer development.^4^

 Beyond prevalence, gaining deeper insight into the factors associated with HPV infection can help clinicians and researchers in controlling the infection. Clinical characteristics, such as age at first sexual intercourse, number of sexual partners, and smoking history, have been linked to HPV acquisition and persistence.^5^ Additionally, the interplay between HPV infection and vaginal microbiome composition remains an area of active investigation. Understanding how factors like bacterial vaginosis influence the susceptibility to HPV infection can inform future preventive interventions.^6^

 Diagnostic tools like Pap smear cytology and HPV molecular testing provide valuable information for cervical cancer screening. Recent advancements in HPV testing technologies offer increased sensitivity and specificity, leading to more accurate risk stratification.^7^ However, the optimal method for HPV testing and Pap smear co-testing remains a topic of debate, particularly with regard to screening intervals for different age groups.^8^

 The present study aimed to contribute to the ongoing dialogue on HPV infection and cervical cancer prevention in Iran. By investigating a cohort of women undergoing gynecological screening, we explored the prevalence and distribution of HPV genotypes and predictors of HPV infection.

## Materials and Methods

 The present retrospective cross-sectional study aimed to investigate the correlation between HPV genotypes and Pap smear findings among women who were referred to the medical laboratory of Academic Center for Education Culture and Research (ACECR), Khorasan Razavi, for a one-year period since January 2023. Among the women who were referred for Pap smear, those who underwent their first cervical screening by Pap smear and did not receive any type of HPV vaccine enrolled after giving an informed consent. Women who had a pregnancy in the past year or had an ongoing pregnancy were not included.

 Cervical cytology samples used for Pap smear analysis were collected during routine gynecological examinations by qualified healthcare providers following standardized protocols using liquid-based cytology. Pap smear results were categorized according to the Bethesda System and a trained cytopathologist reviewed and interpreted the Pap smears according to established guidelines. Viral DNA extraction was performed using the DNA extraction Kit (Simbiolab Company, Mashhad, Iran) and HPV genotyping was performed for 30 HPV genotypes (high-risk genotypes: 16, 18, 31, 33, 35, 39, 45, 51, 52, 56, 58, 59, 66, and 68 and other genotypes including: 4, 6, 11, 26, 42, 43, 44, 53, 54, 61, 62, 67, 73, 82, 89, 90) by HPV genotyping real-time PCR kit (MehrViro, Tehran, Iran) according to the manufacturer’s protocol.

###  Statistical analysis

 Descriptive statistics were used to summarize participant characteristics, HPV genotype prevalence, and Pap smear findings. Chi-square test or Fisher’s exact test was conducted to assess the association between HPV genotype (high-risk vs. low-risk) and Pap smear results. Logistic regression analysis was performed to identify independent predictors of abnormal Pap smears, considering variables such as age, smoking history, and HPV genotype. SPSS version 21.0 was used for all analyses, and a significance level of *P* < 0.05 was considered.

## Results

**Figure 1:**
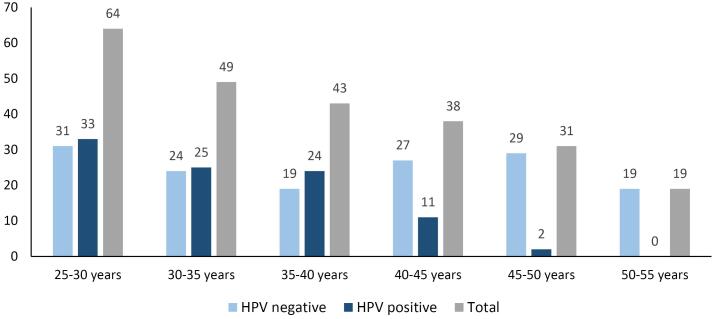
Distribution of age among the study groups based on HPV positivity

**Figure 2:**
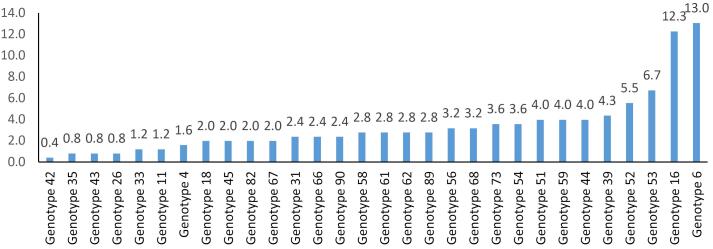
Distribution of HPV genotypes (by percentage) among study population

 A total of 328 women enrolled in this study. The mean ( ± SD) age of the participants was 36 ( ± 11) years. Among the study population, 123 women (37.5%) were diagnosed with HPV infection. Figure 1 demonstrates the age distribution among the study population. Most of the women aged 25-30 years and similarly most of the HPV-positive women were younger than 35 years. While most of the HPV-positive women (60 women) had a single HPV genotype, 29 had 2, 15 had 3, 12 had 4, 5 had 5, 1 had 6, and 1had 11 genotypes. Figure 2 illustrates the prevalence of HPV genotypes among the entire study population. Genotype 6 (33 women) was the most prevalent genotype, followed by genotype 16 (31 women) and genotype 53 (17 women). However, genotype 42 (1 woman) was the least common genotype. Among the study population, most of the women had high risk genotypes (82 women, 25%).

Table 1 demonstrates the study variables among HPV positive (123 women) and negative (205 women) women. The mean age of women with negative and positive results were 39.1 ± 12.1 and 31.5 ± 8.1 years, respectively (*P* = 0.001).

**Table 1:** Demographic information and Pap smear findings among HPV-positive and HPV-negative groups

**Variables**	**Total**		**HPV-negative**		**HPV-positive**		***P *** **Value**
	**Number**	**%**	**Number**	**%**	**Number**	**%**	
Married	183	55.8	114	55.6	69	56.1	0.931
No child	239	72.9	148	72.2	91	74.0	0.683
Chronic illness	54	16.5	39	19.0	15	12.2	0.106
Alcohol consumption	29	8.8	16	7.8	13	10.6	0.393
Smoking	43	13.1	22	10.7	21	17.1	0.099
Endocervical/transformation zone cells	218	66.5	99	48.3	119	96.7	0.001
Tricomonas vaginalis	5	1.5	4	2.0	1	0.8	0.415
Candida infection	45	13.7	20	9.8	25	20.3	0.007
Shift in flora, suggestive of bacterial vaginosis	121	36.9	38	18.5	26	21.1	0.001
Mild inflammation	48	14.6	13	6.3	35	28.5	0.001
Moderate inflammation	66	20.1	25	12.2	41	33.3	0.001
Severe inflammation	39	11.9	12	5.9	27	22.0	0.001
Inflammation	153	46.6	50	24.4	103	83.7	0.001
ASC-US	11	3.4	4	2.0	7	5.7	0.069
Metaplasia	18	5.5	15	7.3	3	2.4	0.060

 HPV-positive women had significantly higher rates of endocervical/transformation zone cells (*P* = 0.001), fungal candida infection (*P* = 0.007), shift in flora suggestive of bacterial vaginosis (*P* = 0.001), and different levels of inflammation (mild, moderate, or severe (*P* = 0.001 for each)). The binary logistic regression model considering these variables demonstrated that the presence of different levels of inflammation, candida infection, suggestive features of bacterial vaginosis, and presence of endocervical/transformation zone cells are the suggestive findings for not having a positive polymerase chain reaction (PCR) test result for HPV (Table 2). Women with endocervical/transformation zone cells had 25% higher odds of having HPV infection (*P* = 0.001, 95% CI: 8.53, 77.69). Moreover, having mild, moderate, and severe inflammation increased the odds of having HPV infection by 14% (*P* = 0.001, 95% CI: 6.18, 36.34), 11% (*P* = 0.001, 95% CI: 5.28, 25.12), and 20% (*P* = 0.001, 95% CI: 7.39, 56.19), respectively.

**Table 2:** Binary logistic regression model for identifying predictors of HPV infection

**Variables**	**HPV+**	**HPV-**	**Crude OR (95% CI)**	***P *** **value**	**Adjusted OR (95% CI)**	***P *** **value**
Transformation zone cells						
No	4	106	Ref.			
Yes	119	99	31.85 (11.33, 89.51)	0.001	25.30 (8.39, 76.20)	0.001
Candida infection						
No	98	185	Ref.	-	-	
Yes	25	20	2.36 (1.24, 4.46)	0.008	1.42 (0.60, 3.35)	0.413
Having children						
No	91	148	Ref.	-	-	
Yes	32	57	0.91 (0.55, 1.51)	0.724	1.65 (0.79, 3.46)	
Mild inflammation						
No	88	192	Ref.	-	-	
Yes	35	13	5.874 (2.96, 11.65)	0.001	14.92 (6.19, 35.95)	< 0.001
Moderate inflammation						
No	82	180	Ref.	-	-	
Yes	41	25	3.50 (2.05, 6.31)	0.001	11.10 (5.11, 24.11)	0.001
Severe inflammation						
No	96	193	Ref.	-	-	-
Yes	27	12	4.523 (2.19, 9.31)	0.001	19.70 (7.16, 54.17)	0.001

*Binary logistic regression was used. The results were not adjusted for other study variables.

## Discussion

 The present study explored HPV prevalence and its association with clinical characteristics among women attending a gynecological clinic. We observed a significant burden of HPV infection, with genotype 16 being prevalent. Interestingly, the presence of endocervical/transformation zone cells and having inflammation were associated with an increased likelihood of HPV positivity, and neither bacterial vaginosis nor candida infection was correlated with HPV infection.

 HPV infection is the most prevalent STI worldwide and it has been estimated that over 80% of sexually active individuals regardless of their age will have at least one HPV infection by the age of 45.^9^ According to the results of our study, most of the women infected with HPV were younger than 35 years old, and more than half of the women aged 25-45 years were infected with the virus. The elevated prevalence of HPV infection among young adults is a complex issue influenced by a multitude of factors and sexual behaviors and practices play a pivotal role; in other words, early sexual initiation, multiple sexual partners, and inconsistent condom use significantly increase the risk of HPV transmission.^10^ A meta-analysis of 78 studies from different regions of Africa, North America, Central America and Mexico, Asia, and Europe reported that most of the HPV infected women were younger than 35 years old, which is similar to our study.^11^ Moreover, while some regions of the world including Europe, Africa, and America face the second peak of infection in women older than 45 years, such peak was not detected among our study population.^11,12^ Factors like access to healthcare, vaccination programs, and sexual behaviors contribute to these variations. It has been reported that specific sexual behaviors, including earlier onset of sexual activity, are linked to a higher risk of HPV infection, and having a higher number of sexual partners increases the likelihood of exposure to HPV.^5^ On the other hand, correct condom use can significantly reduce the risk of HPV transmission among couples.^13^ This may highlight the importance of early education and vaccination programs to target this vulnerable population. While HPV vaccination is among the main preventive strategies in many countries, the distribution of HPV vaccination in every region of Iran has not been clearly studied.^14^ Increasing vaccination coverage, coupled with effective screening programs, is crucial for reducing the burden of HPV-related diseases in the country.^15^ It is essential to recognize that these factors are interconnected, and a comprehensive approach that addresses multiple levels is necessary to effectively reduce HPV infection rates among young adults.

 While over 200 HPV genotypes have been identified, not all genotypes pose the same threat, and high-risk genotypes, particularly 16 and 18, are responsible for most of cervical cancer cases. Understanding these global trends in HPV prevalence is crucial for targeted public health interventions. According to our results, 37.5% of the general population were HPV positive and 25% of the general population had at least one high-risk genotype. A study by de Sanjosé et al evaluating 78 studies including 157 879 women with normal cytology from around the world reported a prevalence of 10.4% for HPV infection, which is much lower compared to our study.^11^ Moreover, Asian studies included in this meta-analysis reported that 8% of the Asian population are infected by the virus. In 2007, the authors estimated that 32% of women carried HPV 16 and HPV 18 worldwide, which is similar to our findings (33% for HPV 16 and 18 infection).^11^ A later systematic review and meta-analysis of 194 studies including 1­016­719 women with normal cytology findings reported a global prevalence of 11.7% for HPV infection, which is lower than our finding. Moreover, they reported that HPV 16, 18, 52, 31, and 58 were the five most common genotypes.^12^ Similarly, other systematic review on Chinese women demonstrated that HPV 16, 18, 58, 52, 33, and 31 were the most common genotypes.^16^ According to our results, HPV 6, 16, 53, 52, and 39 were the five most common genotypes among Iranian women. Among the latest meta-analyses of studies conducted in Asia, the study by Palmer et al reported a prevalence of 15.6% for HPV infection among Japanese women.^17^ A systematic review published in Iran in 2012 reported that the prevalence of HPV infection in healthy women was 7% and HPV genotypes 16, 18, and 31 were the most common genotypes.^18^ More recent studies from Iran showed different results in contrast to such earlier reports. The study conducted by Sabet et al in Khorasan province of Iran reported that 48.4% of male (7.1%) and female (92.9%) individuals are infected with HPV virus and HPV 6, 11, 16, and 53/89 are the most common genotypes, which is more similar to our study.^19^ A similar study recruiting data from 2015-2020 demonstrated that 31.8% of women were HPV positive, which was lower compared to our results. This could be due to the difference in molecular detection method as they used single-step PCR and we considered real-time PCR. Moreover, they reported that the most common low-risk genotype was HPV 6, which was similar to our findings, and the most common high-risk genotype was HPV 31, which was not among the common genotypes in our population.^20^

 One of the main findings of the present study is the absence of the relationship between HPV infection and bacterial vaginosis and candida infection. A recent body of evidence suggested that HPV infection might indirectly promote its own persistence by altering the vaginal microbiome. The study conducted by Lebeau et al suggested that HPV disrupts the natural balance of bacteria in the vagina by reducing the availability of essential nutrients for beneficial *Lactobacillus* bacteria.^21^ In contrast to our study, Lin et al reported that HPV infection is correlated with bacterial vaginosis. Although they reported a lower risk of HPV infection among the general population (16.2%), bacterial vaginosis and HPV 51 and 52 were commonly associated with each other.^22^ A similar study from Africa reported similar findings indicating that HPV infection is significantly associated with bacterial vaginosis.^23^ A study by Guo et al demonstrated that bacterial vaginosis is related to HPV infection and persistent HPV infection is associated with bacterial vaginosis.^24^ On the other hand, some studies such as the study by Romero-Morelos et al indicated that HPV and bacterial vaginosis could be associated with each other but such association is only due to the fact that these bacteria could be a part of cervical microbiome of the Mexican population.^25^ It is noteworthy that the relationship between HPV infection and bacterial vaginosis has been evaluated in patients with chronic illnesses as well. The study conducted by Marques et al demonstrated that among patients infected with immunodeficiency virus, there is no relationship between bacterial vaginosis and HPV infection.^26^

 Considering the study findings regarding the prevalence of HPV genotypes in our country, HPV vaccination still stands as a formidable defense against HPV infection and its associated health crises. By providing effective vaccination against the virus, a robust barrier against a spectrum of cancers, including cervical, anal, and oropharyngeal cancers, will be achieved. This proactive measure not only safeguards vaccinated individuals but also contributes to public immunity, shielding those unable to receive the vaccine. To maximize the impact of HPV vaccination, policymakers should provide accessible and affordable vaccination programs and run public awareness campaigns, which are essential to dispel misconceptions and increase the vaccination rate.

 The cross-sectional design precludes establishing causality between HPV infection and the observed associations. Additionally, the study population was recruited from a single referral laboratory, potentially limiting generalizability. Future research with a larger sample size, a more diverse population, and a prospective design could provide more robust evidence. Moreover, the present study used real-time PCR technique for the detection of HPV infection. Although the technique is considered among accurate molecular techniques for the detection of HPV genotypes, some genotypes may not be covered by this method and false negative results due to low viral load should also be considered in the interpretation of the study results.

 Our findings highlight the need for further investigation into the complex interplay between HPV infection, cervical cytology findings, vaginal microbiome composition, and immune response. Additionally, longitudinal studies can offer valuable insights into the natural history of HPV infection and its progression in this population.

**Highlights**

- The overall HPV prevalence among healthy population is 37.5%.
- Among HPV-positive women, nearly half had a single HPV genotype.
- High-risk HPV genotypes (16 and 18 among all) were identified in one-fourth of HPV-positive women.
- Women with endocervical/transformation zone cells have a 25% higher chance of having HPV infection.
- Neither candida infection nor bacterial vaginosis is related to HPV infection although both of these conditions are more prevalent among HPV positive patients.

## Conclusion

 This study revealed a considerably high prevalence of HPV infection, with genotype 16 being among the most common genotypes. Moreover, the findings highlight the prevalence of HPV infection among women, indicating that a substantial proportion harbor multiple genotypes. The study also revealed an association between HPV status and specific cytological findings. Notably, the presence of endocervical/transformation zone cells and having inflammation, but not bacterial vaginosis and candida infection, were linked to a decreased likelihood of HPV positivity, potentially suggesting a role for these factors in HPV clearance or immune response. Further research is needed to elucidate the underlying mechanisms and causal relationships, ultimately paving the way for more effective prevention and management strategies for HPV-associated cervical cancer.

## Authors’ Contribution

**Conceptualization:** Reza Jafarzadeh Esfehani, Sareh Etemad.

**Data curation:** Amir Mohammad Asghari Baghalan, Bita Naeimi, Shadi Mehrzad, Saina Adib Amin.

**Formal analysis:** Amir Mohammad Asghari Baghalan, Bita Naeimi, Shadi Mehrzad, Saina Adib Amin.

**Investigation:** Amir Mohammad Asghari Baghalan, Bita Naeimi, Shadi Mehrzad, Saina Adib Amin.

**Methodology:** Reza Jafarzadeh Esfehani, Sareh Etemad.

**Project administration:** Reza Jafarzadeh Esfehani, Mohammad Soudyab.

**Resources:** Reza Jafarzadeh Esfehani.

**Software:** Reza Jafarzadeh Esfehani.

**Supervision:** Sareh Etemad.

**Validation:** Mohammad Soudyab.

**Visualization:** Mohammad Soudyab.

**Writing–original draft:** Reza Jafarzadeh Esfehani.

**Writing–review & editing:** Reza Jafarzadeh Esfehani, Mohammad Soudyab.

## Competing Interests

 The authors declare that there is no conflict of interests to declare.

## Ethical Approval

 The present study was approved by the Ethic Committee of Academic Center for Education, Culture, and Research (ACECR), Khorasan Razavi Branch, Mashhad, Iran.

## Funding

 There is no source of funding to declare.
